# Supplementary material for: Complexome profiling of the Chlamydomonas psb28 mutant reveals TEF5 as an early PSII assembly factor
Source: Plant Cell. 2025 Mar 17;37(6):koaf055. doi: 10.1093/plcell/koaf055 (PMC12164590; doi:10.1093/plcell/koaf055)
Supplement: koaf055_Supplementary_Data [file koaf055_supplementary_data.zip › psb28_interactive.zip!/plots/intensity/qualityPlots/peptides/TIG1_Cre01.g001750.t1.2.html]

### Cre01.g001750.t1.2

Trivial name: TIG1  
SeqCoverage [median]: 2.60  
Mass: 60.33 kDa  
  
 

| Sequence | MissedCleavage | Mass [Da] | wtIntensity | muIntensity | Score |
| --- | --- | --- | --- | --- | --- |
| ALEDAVVK | 0 | 843.47 | 0.00 | 3998.39 | 132.09 |
| VTELDKALEDAVVK | 1 | 1528.83 | 0.00 | 674.14 | 33.69 |
|  |  |  | 0.0 | 4672.5 |  |
